# *Vibrio vulnificus* in Taiwan

**DOI:** 10.3201/eid1008.040047

**Published:** 2004-08

**Authors:** Po-Ren Hsueh, Ching-Yih Lin, Hung-Jen Tang, Hsin-Chun Lee, Jien-Wei Liu, Yung-Ching Liu, Yin-Ching Chuang

**Affiliations:** * National Taiwan University Hospital, Taipei, Taiwan;; † Chi-Mei Medical Center, Tainan, Taiwan;; ‡ National Cheng-Kung University Hospital, Tainan, Taiwan;; § Kaohsiung Chang Gung Memorial Hospital, Kaohsiung, Taiwan;; ¶ National Yang-Ming University, School of Medicine, Taipei, Taiwan

**Keywords:** Vibrio vulnificus, Taiwan, emerging, research

## Abstract

Residents in Taiwan are often exposed to marine microorganisms through seafood and occupational exposure. The number of reported cases of infection attributable to this organism has increased since the first case was reported in 1985. The increasing number of cases may be caused by greater disease activity or improved recognition by clinicians or laboratory workers. We analyze a clinical-case series of 84 patients with *Vibrio vulnificus* infection from 1995 to 2000 and describe the molecular epidemiologic features of pathogens isolated from these patients. The spectrum of clinical manifestations and outcomes, options of antimicrobial therapy, and virulence mechanisms were investigated. Results of molecular typing of isolates from humans and marine environment in this country had a high genetic divergence among these isolates. Education and measures are needed to prevent this emerging disease.

## Abstract 2

Clinical features of 84 patients with *V. vulnificus* infection are analyzed and molecular features of isolates are described.

Awareness of *Vibrio vulnificus* as a threat to human health has evolved during the past 30 years (1). In Taiwan, Yuan et al. first reported *V. vulnificus* infection in a patient with septicemia and leg gangrene in Kaohsiung County in 1985 (2). Chuang et al. described an additional 27 cases during a 5-year period from May 1985 to July 1990 and demonstrated three major discernible syndromes: primary septicemia, wound infection, and gastrointestinal diseases. The disease had a high mortality rate (41%) (3). Chuang et al.'s report was also the first to demonstrate the recurrent nature of this disease. Since then, many clinicians and researchers from Taiwan have reported risk factors and the clinical spectrum of this disease on the basis of an increasing number of reported cases (4–12). Many factors have been associated with increased vulnerability of Taiwanese people to *V. vulnificus* infection. These include the high prevalence of hepatitis B or C virus infection-related hepatic diseases (liver cirrhosis and hepatoma), the environment, and the popularity of preparing and eating raw or undercooked seafood (3,13). These factors have drawn considerable interest to finding optimal therapeutic regimens for this infection, as well as to identifying the pathogenesis, ecology, and the reservoirs of this microorganism.

We describe the clinical features of 84 recently identified patients with *V. vulnificus* infection treated from 1995 to 2000 in Taiwan and report the results of molecular typing of 50 isolates of *V. vulnificus* from these patients. We also summarize the recent advances in understanding this newly recognized disease from the Taiwan perspective.

## Disease Prevalence

**Figure 1:**
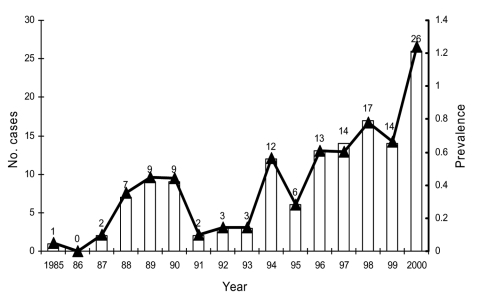
Estimated prevalence (per 10^6^ population) and annual number of cases of *Vibrio vulnificus* infection reported from 1985 to 2000 in Taiwan. The line and triangles represent the prevalence and the bars the number of cases.

Taiwan is a small island situated off the southeast coast of the Asian continent with a population of >22 million people. Figure 1 shows the annual number of reported cases and the estimated prevalence of *V. vulnificus* infection (per 10^6^ persons) from 1985 to 2000 in Taiwan (2–12). Two peaks occurred: one in 1988 to 1990 (0.354–0.450/10^6^ persons) and the other in 1996 to 2000 (0.606–1.237/10^6^ persons). Most reported cases (>90%) occurred in residents of southern Taiwan. In Taiwan, the temperature of surface seawater is usually >18°C, except for February, when it is 17°C–23°C (6). Nearly all cases occurred in the late spring to early fall (April–October), when the seawater temperature is 20°C–29°C. The peak months for infections were June–August (summer season) when the temperature of surface seawater in Taiwan was approximately 26°C–29°C (6).

The reasons for the increased rate of *V. vulnificus* during the past 2 decades are not fully understood. The extent to which the increasing number of cases may be caused by increasing disease activity or improved recognition by clinicians or laboratory workers is also unclear. Since the first report of *V. vulnificus* infection in 1987 and subsequent reports in both humans and environmental studies, clinicians in Taiwan have become increasingly aware of the clinical features of this disease, and laboratory workers more likely to understand how to isolate and identify this pathogen accurately.

## Environmental Habitants and Reservoirs

The occurrence of *V. vulnificus* infections in cultured shrimp and eels has been reported in Taiwan (14). A monthly survey on the distribution of *Vibrionaceae* in seawater from five major harbors in Taiwan was conducted from July 1991 to February 1994 (15). Among the 1,167 *Vibrionaceae* isolates, *V. vulnificus* accounted for 67 (5.7%) (15). This finding indicates that the organism exists autochthonously around the coastal waters or aquatic habitats in Taiwan. Most isolates (91%) from marine water and oysters were indole-negative (biotype I) but some belonged to biotype II (ornithine decarboxylase- and mannitol-positive) (16). Strains of *V. vulnificus* serovar E (also belonging to biotype II) avirulent for eels, which were recovered from water and oysters, were reported (17). Ribotyping analysis of the environmental isolates indicated a great genetic divergence among these isolates (18). More than half of the environmental isolates exhibited virulence in mice, indicating that these isolates might be pathogenic to humans (16). In addition, saline and aqueous ethanol extract (lectins) from some marine algae collected from the northeastern coast of Taiwan had marked antibacterial activity against *V. vulnificus* isolates recovered from the northeastern coast of Taiwan (19). Further study is needed to explore the symbiosis between marine algae and their associated marine vibrios.

## Clinical Features and Outcomes

**Figure 2:**
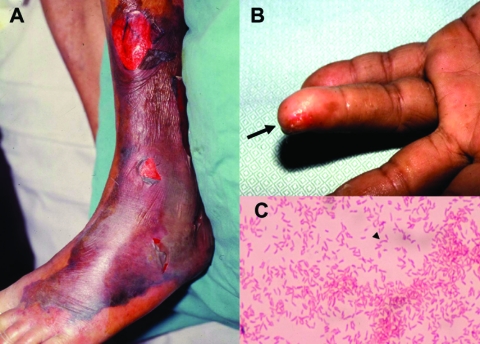
Characteristic skin lesions of *Vibrio vulnificus* infection and morphotype of the microorganism. A) Gangrenous change with hemorrhagic bullae over the leg in a 75-year-old patient with liver cirrhosis in whom septic shock and *V. vulnificus* bacteremia developed. B) *V. vulnificus* bacteremia developed 1 day after a fish bone injury on the fourth finger of the left hand (arrow) in a 45-year-old patient with uremia. C) Gram-negative curved bacilli (arrowhead) isolated from a blood sample of the 45-year-old patient with uremia.

Clinical information from 84 patients *V. vulnificus* infection treated from 1995 to 2000 was obtained from medical records from five hospitals in Taiwan (Table). These hospitals, with a capacities of 1,500 to 2,000 beds, included National Taiwan University Hospital, Taipei; Chi-Mei Medical Center and National Cheng-Kung University Hospital, Tainan; Chang Gung Memorial Hospital-Kaohsiung, Kaohsiung; and Kaohsiung Veterans General Hospital, Kaohsiung. Most of the patients (73%) were male. More than 80% of these patients had various underlying medical conditions with liver disease (particularly hepatitis B or C virus infection-related diseases), which accounted for more than half of the patients, followed by diabetes mellitus and steroid use. Nine patients (16.3%) had exposure to marine injuries (caused by fish or crab bones or eating raw fish) or marine environments (swimming in coastal seawater or raising fish). Although 11 (20%) patients had preexisting skin wounds, exposure of the skin wounds to salt water was not known. More than 60% of these patients had a cutaneous infection, and 50% had necrotizing fasciitis. Approximately three fourths of the patients with necrotizing fasciitis had septic shock. Characteristic cutaneous lesions in patients with necrotizing fasciitis and wounds associated with bacteremia attributable to *V. vulnificus* are shown in Figure 2. Twenty patients (23.8%) had primary septicemia, and 3 were complicated with septic shock.

**Table:** Clinical characteristics of 84 patients with *Vibrio vulnificus* infections who were treated at five major hospitals, Taiwan, 1995–2000

Characteristic (no. of patients for whom information was available)	No. of patients (%)
Sex (n = 84)	
Male/female	61 (72.6)/23 (27.4)
Age, mean/range (yr)	60/9-87
Underlying disease (n = 84)^a^	
Chronic hepatitis B or C virus infection	10 (11.9)
Liver cirrhosis	35 (41.7)
Hepatitis B or C virus infection-related	21
Alcoholic	7
Hepatoma	7
Diabetes mellitus	13 (15.5)
Steroid use	10 (11.9)
Alcoholism	8 ( 9.5)
Renal insufficiency	6 (7.1)
Other malignancies	3 (3.6)
None	12 (14.3)
Type of infection (n = 84)	
Cutaneous infection	57 (67.9)
Cellulitis	15 (17.9)
With bacteremia	5
With septic shock	6
Necrotizing fasciitis	42 (50.0)
With bacteremia	2
With septic shock	32
Primary septicemia	20 (23.8)
With septic shock	3
Spontaneous bacterial peritonitis	6 ( 7.1)
Meningitis	1 ( 1.2)
Exposure history (n = 55)	
Injury from handling marine animals (fish, crab)	7 (12.7)
Preexisting skin wounds	11 (20.0)
Ingestion of raw seafood	2 ( 3.6)
None	35 (63.6)
Initial antibiotic treatment (n = 82)	
A third-generation cephalosporin^b^ plus minocycline	38 (46.3)
A first-generation cephalosporin plus an aminoglycoside	15 (18.3)
Other combinations^c^	29 (35.4)
Surgical treatment (cutaneous lesions, n = 57)	
Incision and drainage, débridement and/or fasciotomy	43 (75.4)
Amputation	6 (10.5)
Outcome	
Survived	57 (67.9)
Died	25 (29.8)
Unknown	2 ( 2.4)

^a^Patients might have more than two underlying diseases. ^b^Includes ceftazidime, cefotaxime, ceftriaxone, and moxalactam. ^c^Includes a penicillin or a first-generation cephalosporin plus an aminoglycoside or minocycline.

Similar to the previous findings, we found no patients with gastroenteritis caused by to *V. vulnificus* (3). Most patients with gastroenteritis or diarrheal illness in Taiwan do not seek care at the large teaching hospitals; they also do not usually have a stool culture, which might explain the lack of patients with gastrointestinal illness attributable to *V. vulnificus*.

**Figure 3:**
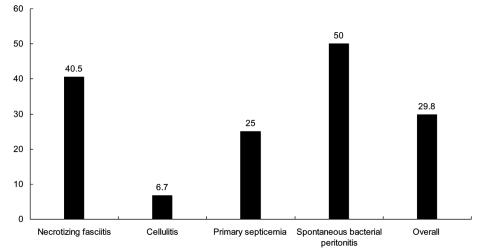
Rates of deaths according to different types of infection of 84 patients with *Vibrio vulnificus* infection.

A third-generation cephalosporin plus minocycline was used as the definite treatment regimen in 46% of patients. Among 57 patients with cutaneous lesions, 49 (86.0%) had some form of surgical treatment (incision and drainage, débridement, fasciotomy, and amputation). The overall case-fatality rate was approximately 30% (Figure 3), which was similar to that reported previously among patients seen from 1995 to 1990 (3). Patients with spontaneous bacterial peritonitis had the highest case-fatality rate (50%), followed by necrotizing fasciitis (40.5%). Patients with cellulitis had the lowest case-fatality rate (6.7%).

## Antimicrobial Drug Resistance and Treatment Options

MICs were determined and interpreted by using the MIC interpretive criteria for *Enterobacteriaceae* recommended by the National Committee for Clinical Laboratory Standards (20–23). All isolates of *V. vulnificus* in Taiwan, which were collected from the previous studies, were susceptible to the following agents (MIC_90_): ampicillin (1 µg/mL), carbenicillin (4 µg/mL), cephalothin (4 µg/mL), cefamandole (2 µg/mL), cefotaxime (<0.03–0.06 µg/mL), ceftriaxone (<0.03 µg/mL), cefoperazone (0.12 µg/mL), aztreonam (8 µg/mL), imipenem (<0.03–0.12 µg/mL), gentamicin (4 µg/mL), amikacin (8 µg/mL), tetracycline (0.25 µg/mL), minocycline (0.06–0.25 µg/mL), chloramphenicol (0.5 µg/mL), and fluoroquinolones: ofloxacin (<0.03 µg/mL), lomefloxacin (0.12 µg/mL), ciprofloxacin (<0.03–0.03 µg/mL), levofloxacin (0.03 µg/mL), moxifloxacin (0.06 µg/mL), gatifloxacin (0.06 µg/mL), and sparfloxacin (0.06 µg/mL) (20–23). Few isolates were not susceptible to ceftazidime (MIC 32 µg/mL) and moxalactam (MIC 32 µg/mL) (21). All isolates were resistant to clindamycin (MICs >256 µg/mL) (20). In vitro synergism between cefotaxime and minocycline against *V. vulnificus* isolates was documented by time-kill study (21). Time-kill study also demonstrated that fluoroquinolones at concentrations of two times the MIC had a persistent inhibitory effect on *V. vulnificus* for >48 hours (23).

In vivo study using a mouse model of *V. vulnificus* infection clearly indicated that combination therapy with cefotaxime and minocycline is distinctly superior to therapy with cefotaxime or minocycline alone (22). A similar effect of newer fluoroquinolones as single agents compared with the cefotaxime-minocycline combination was also demonstrated in the treatment of severe experimental *V. vulnificus* infection (23).

On the basis of the in vitro and in vivo animal studies, along with clinical outcome analysis, combination therapy with cefotaxime (2 g every 6 h intravenously) and minocycline (100 mg every 12 h intravenously) was recommended for treating adult patients with bacteremia and severe soft-tissue infection caused by *V. vulnificus* (21,22). For severe soft-tissue infection (necrotizing fasciitis, tissue necrosis with gangrene change, and myositis), early and aggressive surgical interventions (incision and drainage, débridement, fasciotomy, and amputation) are important in saving the life of the patient.

## Pathogenesis

More than 90% of *V. vulnificus* isolates whose biotypes were determined belonged to biotype I, which is well known to be pathogenic for humans (15,16). In 1997, Chuang et al. first demonstrated that severe damage of the connective tissue of a mouse by *V. vulnificus* wound infection could be mediated by a recombinant extracellular metalloprotease (able to digest collagen and elastin) (24). Lee et al. also illustrated that extracellular products of *V. vulnificus* were lethal to fish (moribund black porgy, *Acanthopagrus* *schlegeli*) (25). Genes (*vvp* and *empV*) encoding the metalloprotease and gene (*vllY*) encoding a novel hemolysin of *V. vulnificus* were subsequently cloned and characterized (26–28).

Hor et al. showed that isogenic protease-deficient (PD) mutant of *V. vulnificus* was as virulent as its parent strains in mice infected intraperitoneally and was 10-fold more virulent in mice infected through the oral route (29). A metalloprotease- and cytolysin-deficient mutant of *V. vulnificus* also had similar virulence in mice, and its cytotoxicity for HEP-2 cells (cytotoxin) compared with those of the wild-type strains (30). These findings suggest that neither metalloprotease nor cytolysin is essential for the virulence or invasiveness of *V. vulnificus* in mice. A possible multifactor interaction in bacterial virulence might be present but to an extent that is not yet clear. However, two genes, *vvn* (encoding a periplasmic nuclease, *Vvn*) and *smcR* (encoding *SmcR*, which regulate metalloprotease gene expression), were not required for *V. vulnificus* virulence in mice (31,32).

Animal studies clearly demonstrated that iron could increase the growth rate of *V. vulnificus*, which quickly reached a lethal concentration with enhanced cytotoxicity in the iron-overloaded mice (33). A study of the survival of *V. vulnificus* in whole blood from patients with different degrees of liver disease showed that high serum ferritin levels and low phagocytosis activity of neutrophils were independent and important predictors of survival of the organism in blood (34). These findings indicated that patients with chronic hepatitis, liver cirrhosis, and hepatoma (high serum ferritin levels and lower phagocytosis) were at high risk for *V. vulnificus* infection (34). Although many putative virulence factors have been studied for this exceptionally virulent human pathogen in Taiwan, how these factors and other veiled factors (such as capsular polysaccharide and lipopolysaccharide) interact to produce dramatic infections and what host aspects (such as overproduction of proinflammatory cytokines) are essential to infection are yet to be elucidated (3).

## Molecular Epidemiologic Features

Results of molecular typing by using restriction fragment length polymorphism analysis of rRNA (ribotyping) among 13 clinical and environmental (from seawater and eels in southern Taiwan) isolates of *V. vulnificus* and arbitrarily primed polymerase chain reaction analysis of 37 isolates (24 clinical isolates and 13 from seawater from coast areas around Taiwan) were previously reported (18,35). Both showed high genetic divergence among clinical and environmental isolates.

The concentration of *V. vulnificus* in recent clinical and environmental isolates in southern Taiwan indicates the possibility of clonal spread in this area. In this study, 50 isolates of *V. vulnificus* collected from 1995 to 2000 from southern (46 isolates) and northern (4 isolates) Taiwan were analyzed. These isolates included those from various clinical specimens (blood and wound pus) of 50 patients with *V. vulnificus* infection. All isolates of *V. vulnificus* were identified by using conventional methods and the O/129 susceptibility tests. Identification of the isolates was further confirmed by the API 32 GN system (bioMérieux Vitek, Inc., Hazelwood, MO). Pulsed-field gel electrophoresis (PFGE) analysis was performed by a method described previously by Tenover et al. (36,37). DNA was digested by the restriction enzymes *Sfi*I and *Not*I (Promega, Madison, WI). All isolates were not identical in PFGE profiles (50 pulsotypes were found), and only two isolates from southern Taiwan were closely related (within three bands of difference). These findings support the high degree of heterogeneity among isolates of *V. vulnificus* that cause human infections in Taiwan.

## Preventive Measures

Residents of Taiwan, particularly those with preexisting liver and other chronic, underlying medical conditions (renal disease, diabetes mellitus, chronic steroid use), should be educated in measures to prevent acquiring *V. vulnificus* infections. This bacterium is present in warm coastal waters around Taiwan during the summer months, particularly in the southern region. Exposing open wounds or broken skin to warm salt or brackish water or to raw marine animals harvested from such waters should be avoided. Patients at high risk should wear protective clothing (e.g., gloves) when handling seafood (fish, oysters, clams, shrimp, eels, and other shellfish) and not eat raw or improperly cooked seafood. Because this disease is rapidly progressive and deadly if not recognized promptly and treated aggressively, any illness (such as fever or skin lesions), which develops in patients at risk after contact with marine animals or waters or ingestion of seafood requires immediate medical care.

The government in Taiwan (Department of Health and Council of Agriculture) should encourage food companies to put warning labels on seafood containers, menus, and public health brochures. The wording of such labeling should be similar to the label required by the Florida Department of Natural Resources for all wholesale shell food and shucked products: "Consumer Information—There is a risk associated with consuming raw oysters or any raw animal protein. If you have chronic illness of the liver, stomach, or blood or have immune disorders, you are at a greater risk of serious illness from raw oysters and should eat oysters fully cooked. If unsure of your risk, consult a physician" (38).

## Conclusion

Residents of Taiwan have a high prevalence of chronic liver disease and are often exposed to marine microorganisms present in the sea that surrounds the island or rivers, lakes, or ponds inside the island. The presence of high genetic divergence among *V. vulnificus* isolates from humans and the environment indicates that this virulent bacterium is ubiquitous in nature. When *V. vulnificus* is suspected as the cause of sepsis, empiric therapy that includes a third-generation cephalosporin and minocycline should be administered. It should be standard practice for physicians to advise patients with underlying medical illness to avoid eating raw or undercooked seafood and to avoid exposing wounds to seawater.
